# *FcLDP1*, a Gene Encoding a Late Embryogenesis Abundant (LEA) Domain Protein, Responds to Brassinosteroids and Abscisic Acid during the Development of Fruits in *Fragaria chiloensis*

**DOI:** 10.3389/fpls.2016.00788

**Published:** 2016-06-14

**Authors:** Analía Espinoza, Rodrigo Contreras, Gustavo E. Zúñiga, Raúl Herrera, María Alejandra Moya-León, Lorena Norambuena, Michael Handford

**Affiliations:** ^1^Centro de Biología Molecular Vegetal, Departamento de Biología, Facultad de Ciencias, Universidad de ChileSantiago, Chile; ^2^Facultad de Química y Biología, Universidad de Santiago de ChileSantiago, Chile; ^3^Centro para el Desarrollo de la Nanociencia y la Nanotecnología, Universidad de Santiago de ChileSantiago, Chile; ^4^Laboratorio de Fisiología Vegetal y Genética Molecular, Instituto de Ciencias Biológicas, Universidad de TalcaTalca, Chile

**Keywords:** abscisic acid, brassinosteroids, *Fragaria chiloensis*, fruit development, LEA domain protein, non-climacteric

## Abstract

White Chilean strawberries (*Fragaria chiloensis*) are non-climacteric fruits, with an exotic color and aroma. In order to discover genes involved in the development of these fruits, we identified a fragment of a gene encoding a late embryogenesis abundant domain protein, *FcLDP1*, that was expressed in early stages of fruit development, particularly in receptacles. Hormones play key roles in regulating the development of non-climacteric fruits. We show that the brassinosteroid content of the white strawberry varies during development. Additionally, *FcLDP1* as well as the closest ortholog in the woodland strawberry, *F. vesca* (*FvLDP1*) possess multiple brassinosteroid, as well as abscisic acid (ABA) response motifs in the promoter region, consistent with the response of transiently expressed *FcLDP1* promoter-GFP fusions to these hormones, and the rise in *FcLDP1* transcript levels in white strawberry fruits treated with brassinosteroids or ABA. These findings suggest that both hormones regulate *FcLDP1* expression during the development of white strawberries.

## Introduction

The octoploid white Chilean strawberry, *Fragaria chiloensis* produces exotic fruits with excellent taste, aroma, and color, and it is a parental species of the commercial red strawberry, *F.*× *ananassa* (Folta et al., 2005). The genus *Fragaria* develops aggregate accessory fruits with hundreds of achenes attached to a fleshy receptacle. Receptacles are composed of about 90% water and 10% soluble solids, substances which are imported from distal parts of the plant. As storage sinks, fruits are thus highly dependent on the xylem and phloem which together make up the vascular system. For this reason, the development of the vascular system is essential for fruit development, determining the texture and integrity of the mature fruit (Aharoni et al., 2002).

During the development of fruits, plant hormones guide multiple changes in physiological and biochemical processes (McAtee et al., 2013). In climacteric fruit, such as apples (*Malus domestica*) and tomatoes (*Solanum lycopersicum*), ethylene is the key hormone that regulates the ripening process (Cherian et al., 2014). However, the control of ripening in non-climacteric fruits, such as *F. chiloensis* (Aharoni et al., 2002) is less well understood. Final fruit size is determined by rapid initial cell division in early stages of development, and then by cell expansion, as water and other nutrients accumulate inside the cells (Rosli et al., 2004). During the early stages of development of both climacteric and non-climacteric fruits, changes in the levels of auxin, gibberellins, ABA, cytokinins, and brassinosteroids have been reported (Mounet et al., 2009; Symons et al., 2012). It has been observed that auxin gradients are essential for growth, and several genes, such as *expansin*, *endo-xyloglucan*, and *pectate lyase* families have been shown to be regulated by either auxins, gibberellins, or both (Catalá et al., 2000; Chen et al., 2002). In red strawberry (*F.* × *ananassa*), ABA levels rise gradually throughout fruit development and in the ripening process (Symons et al., 2012), consistent with the observation that down-regulation of an ABA receptor, FaPYR1, delays red strawberry fruit ripening (Chai et al., 2011). There are greater cytokinin levels after pollination, which favor the proliferation of ovary tissues, while brassinosteroids have been described as participating in elongation and cell division, vascular differentiation, flowering, and pollen development (Li, 2010). Nevertheless, to date the presence and potential effects of brassinosteroids have not been studied during the early stages of development of *F. chiloensis*.

Brassinosteroids and brassinosteroid signaling have been studied in *Arabidopsis thaliana*, based on the identification and analysis of mutants. The kinase receptor BRASSINOSTEROIDS INSENSITIVE1 (BRI1) activates transcription on recruitment of the co-receptor kinase BRI1-ASSOCIATED RECEPTOR KINASE1 (BAK1; Li et al., 2002). The Brassinazole-Resistant1 proteins (BZR1), bri1-EMS-Suppressor1 (BES1), and interacting BES1 MYC-like 1 (BIM) are transcription factors that regulate the brassinosteroid response (He et al., 2002; Yin et al., 2005; Jiang et al., 2015). In non-climacteric cucumbers (*Cucumis sativus*), brassinosteroids play a role in fruit set and development, as exogenous applications trigger development of unfertilized ovaries, and blocking perception of brassinosteroids with brassinazole prevents the development of parthenocarpic fruit (Fu et al., 2008). Also, in red strawberries the *BRI1* transcript level is greater during the first stage of development, and brassinazole application prevents coloration (Chai et al., 2013).

Considering the role of brassinosteroids in non-climacteric fruit development, the aim of this study was to search for genes in white strawberry that could be regulated by this hormone. To do so, we used results from a suppression subtractive hybridization (SSH) library that contains candidate genes that are differentially expressed during development and ripening of *F. chiloensis* fruits (Pimentel et al., 2010). We selected a gene encoding a late embryogenesis abundant (LEA) domain protein, *FcLDP1*, as it was the most highly represented gene in the SSH library in the mid-stages of fruit development. *FcLDP1* possesses brassinosteroid response elements (REs) in its promoter, and responds to exogenous applications of this hormone, as well as ABA, both when heterologously expressed in tobacco (*Nicotiana tabacum*) and also in white strawberry fruits.

## Materials and Methods

### Plant Material

Fruit of *F. chiloensis* cultivated in the same commercial field were obtained from Purén, Araucania region, Chile (latitude 38°.04′8.6′′S; longitude 73°.14′2.96′′W) in December 2014. Four developmental stages were considered, as reported in Figueroa et al. (2008), based on the size and color of the receptacle and achenes: C1, small fruits with a green receptacle and green achenes; C2, large fruits with a green receptacle and red achenes; C3, turning stage with a white receptacle and red achenes; and C4, ripe fruits with a pink receptacle and red achenes. Fruits in the laboratory were treated with various hormones and immediately frozen at -80°C until analysis.

Seeds of tobacco were germinated and grown under standard greenhouse conditions (16 h light/8 h dark, room temperature, 60–70% relative humidity) and watered with Murashige and Skoog nutrient solution (Phytotechnology Laboratories, USA). Leaves of 6-week-old plants were used to perform transient expression assays.

### Hormonal Treatment

Four strawberry fruits for each replicate with three biological replicates for each time point were treated with the following hormonal compounds: 1 mM ABA (Sigma), 1 mM 1-NAA (auxin; Sigma), 10 μM 24-epibrassinolide (brassinosteroid; Sigma), 2 g/L Ethrel (ethylene, Bayer CropScience) or 100 μM methyl jasmonate (Sigma). All compounds were dissolved in ethanol, and the corresponding buffer-only controls were also performed. Fruits at the C2 developmental stage were placed in a plastic box and immersed in citrate buffer containing each hormone, and then collected at different time points post-treatment. All samples were frozen in liquid nitrogen and stored at -80°C.

### DNA and RNA Isolation

DNA and RNA samples were isolated from each developmental stage, using the CTAB method with modifications (Chang et al., 1993; Porebski et al., 1997). RNA samples were treated with RNase-free DNase I (Thermo Fisher Scientific) to remove contaminant genomic DNA. The integrity and concentration of isolated DNA and RNA were measured in a NanoVue Plus Spectrophotometer (Nanodrop Technologies, GE Healthcare, UK). First-strand cDNAs from each developmental stage were synthesized from total RNA (2 μg) using the ImProm-II^TM^ Reverse Transcription System (Promega), according to the manufacturer’s instructions.

### Cloning of *FcLDP1* Promoter and cDNA

A fragment of *FcLDP1* was obtained from an SSH library constructed from *F. chiloensis* fruits at different developmental stages (Pimentel et al., 2010). To obtain the full-length cDNA sequence of *FcLDP1*, primers were designed from the orthologous sequence of *Fragaria vesca* (NC_020496.1, LG6:14021042-14026562; equivalent to gene15289, which we named *FvLDP1*)^1^. The cDNA sequence was amplified by PCR using Fv-LDPf and Fv-LDPr from a mixture of *F. chiloensis* fruits at different development stages and cloned in pCR8-TOPO (Invitrogen). Subsequently, the promoter of *FcLDP1* was amplified and cloned in pCR8 from genomic DNA, using primers designed from the orthologous sequence in *F. vesca* (forward primer: Fv-pLDPf) and complementary to the cloned *FcLDP1* cDNA sequence (reverse primer: Fc-pLDPr). All primer sequences are shown in Supplementary Table S1.

### *In silico* Analyses

All cDNA and promoter clones were sequenced and analyzed by BLAST in order to assess their similarity and identity to orthologous sequences^1,^^2^. The transcriptional start site, TATA-box, CAAT-box, and putative *cis*-acting elements were identified using the Plant Pan Database^3^. The phylogenetic tree was constructed using http://phylogeny.lirmm.fr/phylo_cgi/simple_phylogeny.cgi.

### Real-Time PCR Expression Analysis

The transcript levels of *FcLDP1* in different stages of fruit development, and after hormone treatments were determined by RT-qPCR, based on the protocol described in Aguayo et al. (2013). Specific primers were designed with the Primer 3 software^4^ (Supplementary Table S1). Analysis by RT-qPCR was carried out using 10 μL SensiMix SYBR Hi-ROX kit (Bioline), with 5 μM of each primer, in a Stratagene MX3000P (Agilent Technologies) with the following parameters: 95°C for 10 min, then 40 cycles of 95°C for 15 s, 60°C for 15 s, and 72°C for 20 s. *FvGAPDH* (Pimentel et al., 2010) and *FcRIB314* (Amil-Ruiz et al., 2013) were used as constitutively expressed control genes. The specificity of the amplification products was confirmed by registration of the melting curve of the PCR products, a heat dissociation protocol (from 55 to 95°C) and visualization in 3% agarose gels. The *Ct* values obtained (threshold cycle value) were analyzed by averaging the three biological replicas and two technical replicas of each sample (Livak and Schmittgen, 2001).

### Measurement of *FcLDP1* Promoter Activity

The promoter of *FcLDP1* cloned in pCR8 was recombined into pKWSF7.0 (Karimi et al., 2002). *Agrobacterium tumefaciens* strain EHA105 was transformed by heat shock and a liquid culture was grown for 24 h at 28°C. Cells where then pelleted (4500 × *g* for 10 min) and resuspended in 10 mM MgSO_4_, 10 mM MES, and pH adjusted to 5.6, to reach a final OD_600__nm_ of 0.5. Young leaves of 6-week-old tobacco plants were manually infiltrated with each bacterial suspension through the abaxial surface using a needleless plastic syringe. Transient GFP expression was evaluated by a Zeiss LSM510 Confocal, and images were processed using ImageJ, 3 days post-infiltration (Utz and Handford, 2015).

### Determination of Brassinosteroid Concentrations

The extraction and measurement of brassinosteroid concentrations were performed by the protocol described by Pan et al. (2010). Twenty microliters of white strawberry fruit extract was resolved in a reverse-phase C_18_ Gemini LC-MS 1200s-6410 triplequad Agilent HPLC-ESI-MS/MS system (Palo Alto, CA, USA). Commercial epibrassinolide (Sigma) was used as a positive control.

### Statistical Analyses

The statistical significance of the differences of the mean data between treatment groups was calculated by analysis of variance and by Student’s *t*-test. Results were considered statistically significant when *p* ≤ 0.05 (Tukey test). All analyses were performed with GraphPad Prism 5.0 (GraphPad, San Diego, CA, USA).

## Results and Discussion

**FIGURE 1:**
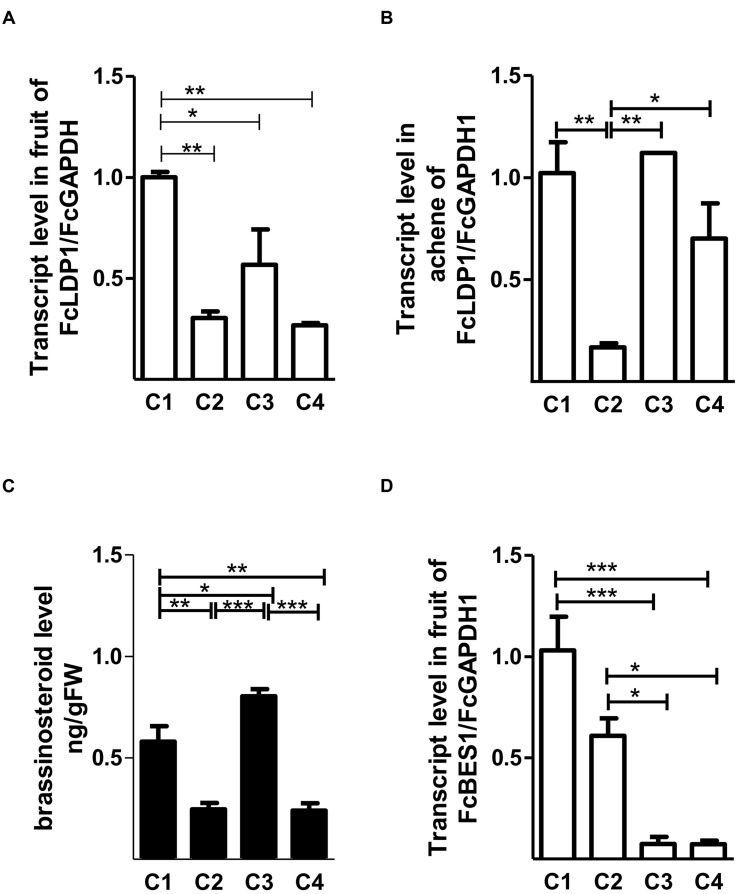
***FcLDP1* transcript and brassinosteroid levels in *Fragaria chiloensis*.** The relative transcript levels of *FcLDP1* at different stages of white strawberry development in whole fruits **(A)** and achenes **(B)**. Endogenous levels (ng/g^-1^ FW) of epibrassinolide during fruit development **(C)**. The relative expression levels of the brassinosteroid marker gene *FcBES1* during fruit development **(D)**. The error bars show standard errors, and asterisks indicate statistical differences with one-way ANOVA and Tukey tests (*p < 0.05; **p < 0.01; ***p < 0.001).

The development of *F. chiloensis* fruits has been divided into four stages according to the size and color of achenes and receptacles (Figueroa et al., 2008). Specifically, these are small green fruits with green achenes (C1); large green fruits with red achenes (C2); white fruits with red achenes (C3), and pink fruits with red achenes (C4). An SSH library from cDNA of *F. chiloensis* collected at the four developmental stages was generated in order to better understand the processes underlying the development and ripening of the Chilean white strawberry (Pimentel et al., 2010), and the results analyzed in detail (Handford et al., 2014). Considering the changes in color, firmness and size in white strawberry fruits, and elevated brassinosteroid levels before the ripening of non-climacteric fruits, we focussed on those transcripts that were more abundant in C3 compared to C2 fruit stages. Altogether, 54 ESTs representing 11 contigs were differentially expressed between these two stages (Pimentel et al., 2010). We selected by far the most highly represented contig, SSHC-051 (represented by 25 of the 54 ESTs) for future analysis. Consistent with the premise that it may respond to brassinosteroids, the corresponding orthologous gene in the most closely related species to *F. chiloensis*, the woodland strawberry *F. vesca* (*FvLDP1*, Shulaev et al., 2011) possesses six elements that respond to brassinosteroids in its promoter. Due to the absence of a genome sequence for *F. chiloensis*, for cloning SSHC-051 we designed primers using the coding sequence of NC_020496.1. We determined that the coding region of the gene in *F. chiloensis* was 693 bp in length (Genbank accession number: KX246933), and that the corresponding genomic DNA lacked introns (Supplementary Figure S1). The predicted protein sequence was 52.2 and 41.1% identical to AtNDR1/Hairpin-induced protein (HIN1)-like 10 and AtSYP24 of *A. thaliana*, respectively. AtNDR1/HIN1-like 10 plays a crucial role in pathogen-induced plant responses to biotic stress (Zheng et al., 2004), whereas syntaxins of the SYP2 family are responsible for protein trafficking between pre-vacuolar compartments (PVC) and vacuoles (Sanderfoot et al., 2000; Shirakawa et al., 2010). Also, and like AtNDR1/HIN1-like 10 and AtSYP24, the sequence encoded by SSHC-051 possesses a LEA 14 (IPR004864) domain (Supplementary Figure S2), which is believed to be involved in abiotic stress tolerance and is characterized by accumulation in the late stages of embryogenesis in *Arabidopsis* (Hundertmark and Hincha, 2008) and in climacteric species such as peach (*Prunus persica*; Bonghi et al., 2011). However, in non-climacteric species like *F. chiloensis*, their role is more poorly understood. Considering the presence of LEA 14, we named the protein encoded by SSHC-051 as FcLDP1. Orthologs of *FcLDP1* are present in the genomes of a wide diversity of plant species, including *F. vesca* (*FvLDP1*), *N. tabacum*, and the African oil palm (*Elaeis guineensis*; Supplementary Figure S3). With the aim of determining the expression profile of *FcLDP1* throughout fruit development, RT-qPCR analysis was performed using fruits from the 2014 season. The transcripts of this gene were more abundant in whole fruits during the C1 stage (**Figure 1A**). In achenes, the transcript levels of *FcLDP1* were higher in C1 and C3 stages (**Figure 1B**), indicating that the encoded protein may play a role in different stages of fruit and embryo development. Interestingly, *FvLDP1* is also expressed during *F. vesca* fruit development. Highest levels of *FvLDP1* expression are found in tissues from the achenes compared to receptacles in early stages of development (named as gene15289 in Kang et al., 2013), and while transcript levels of *FcLDP1* between achenes and receptacles cannot be compared, greatest accumulation of *FcLDP1* is found in achenes at the C1 stage, roughly equivalent to those *F. vesca* fruits examined by Kang et al. (2013). After dissecting *F. vesca* achenes, the maximum levels of expression are present in ovary wall tissues, which subsequently develop into the pericarp in mature fruits (Kang et al., 2013).

Brassinosteroids are one of the hormones that are more abundant in the early stages of fruit development. This hormone belongs to a steroidal group of plant hormones, essential for normal plant development (Kauschmann et al., 1996). Exogenous brassinosteroid application promotes ripening in climacteric and non-climacteric fruits (Vidya Vardhini and Rao, 2002; Symons et al., 2006). Changes in levels of brassinosteroids can modulate maturation in conjunction with auxin, ABA, and other hormones (Symons et al., 2012). In white strawberry fruit, brassinosteroid levels peak at the C1 and C3 stages (**Figure 1C**). High levels in C1 may be related to the known role of brassinosteroids in promoting cell division, thus affecting the final size of the fruit, as shown when cucumbers are treated with exogenous applications of 24-epibrassinolide (Fu et al., 2008). On the other hand, the increase during the C3 stage may favor maturation, which is supported by the fact that ripening of *F. × ananassa* is accelerated when immature large green fruits (C2-equivalent) are exposed to brassinosteroids (Chai et al., 2013). Blocking the perception of brassinosteroids by using brassinazole generates a delay in maturation in *F. × ananassa* (Chai et al., 2013). Consistent with the high transcript levels of *FcLDP1* during the C1 stage of fruit development of *F. chiloensis*, a greater accumulation of the transcript levels of the brassinosteroid transcriptional stimulator *FcBES1* was found in C1 (**Figure 1D**). Furthermore, in whole fruits, levels of *FcBES1* decreased progressively toward the C4 stage (**Figure 1D**), as did *FcLDP1* levels (**Figure 1A**). Nevertheless, it is possible that other transcription factors regulate brassinosteroid responses in the C3 stage when a high content of brassinosteroid was detected and *FcBES1* transcript levels were low. Considering the biological processes regulated by the BES1 transcription factor (Sun et al., 2010), it is possible that FcLDP1 is involved during the early stages of fruit development, when cell division rates are high.

**FIGURE 2:**
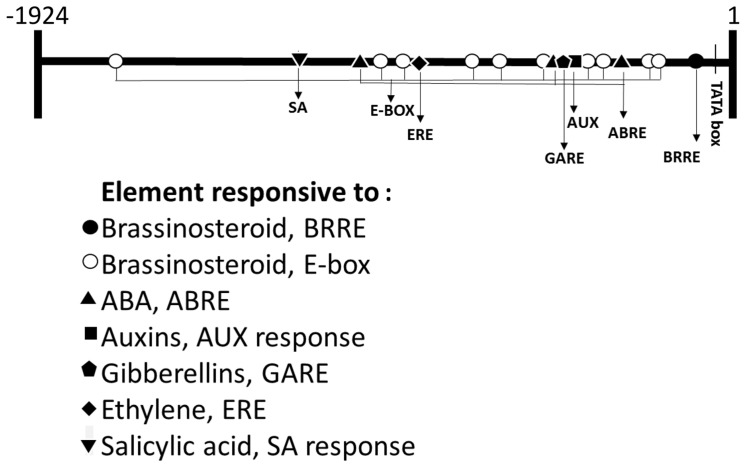
***In silico* analysis of the promoter of *FcLDP1*.** The promoter and gene sequence of *FcLDP1* were amplified, cloned, and the prediction of transcriptional start site, TATA-box, and hormonal response elements (REs) was performed using the Plant Pan Database (plantpan2.itps.ncku.edu.tw).

With the purpose of determining whether brassinosteroids control *FcLDP1* expression, the *FcLDP1* promoter region was obtained using a forward primer designed against the equivalent orthologous region approximately 2000 bp upstream of the transcription initiation sequence in *F. vesca* (*FvLDP1*), and a reverse primer in the 5′ coding sequence of *FcLDP1*. Consequently, a 1924 bp putative promoter sequence of *FcLDP1* was amplified and cloned. We verified that the promoter and cDNA sequences were indeed contiguous, by amplifying *F. chiloensis* genomic DNA. A fragment of the expected size (approximately 2600 bp; Supplementary Figure S4) was obtained. *In silico* analysis of the promoter sequence revealed the presence of a TATA box -111 bp upstream of the predicted start site of transcription (Genbank accession number: KX246932). Furthermore, multiple sequences associated with regulatory hormone binding sites, as well as light REs were present (**Figure 2**) which may be involved in controlling reproductive development. Of the brassinosteroid responsive transcription factors, BZR1 binds to brassinosteroid response elements (BRRE, CGTGYG; He et al., 2005) while BIM/BES1 binds to E-boxes (CANNTG), driving expression of target genes (Yin et al., 2005). *In silico* analysis of the putative promoter sequence of *FcLDP1* shows that 1 BRRE (He et al., 2002) and 10 E-boxes (He et al., 2005) are present, suggesting that *FcLDP1* expression is controlled by this hormone. Furthermore, we found putative REs to auxin, gibberellin, ABA, and salicylic acid (**Table 1**; Gubler and Jacobsen, 1992; Itzhaki et al., 1994; Shen and Ho, 1995; Shah and Klessig, 1996; Guilfoyle et al., 1998). Moreover, putative binding sites for transcription factors described in floral development [SEP4 AGL3 (Liu et al., 2013); AG and ATH-1 (Li et al., 2012; Ó’Maoiléidigh et al., 2013)], synthesis of lignin [LIM1, (Kawaoka et al., 2000; Kawaoka and Ebinuma, 2001)], flavonoid synthesis (Corn P site, Zhang et al., 2000) and a bellringer transcription factor site associated with vascular differentiation (Etchells et al., 2012) are also contained within the *FcLDP1* promoter. Using data from the re-annotation of the *F. vesca* genome (Darwish et al., 2015), we analyzed the equivalent putative promoter region of the closest ortholog to *FcLDP1*, *FvLDP1* (2.0 kb; **Table 1**). While REs to most hormones are present in both species, fewer elements that respond to brassinosteroids (2 BRRE and 4 E-boxes in *FvLDP1*; 1 BRRE and 10 E-boxes in *FcLDP1*) and fewer ABA response elements (1 ABRE in *FvLDP1* and 3 ABRE in *FcLDP1*) are present in the woodland strawberry compared to the white Chilean strawberry (**Table 1**). These differences may indicate subtle variations in the hormone responsiveness that modulate the expression of *LDP1* during reproductive strawberry development.

**Table 1:** Predicted hormone response elements in the *FcLDP1* and *FvLDP1* promoters.

Hormone	Binding	Number		Position (bp upstream of putative start codon)	
	site	*Fc*	*Fv*	*F. chiloensis*	*F. vesca*
Brassinosteroid	BRRE	1	2	-108	- (145, 295)
	E-box	10	4	-(235, 264, 445, 542, 630, 849, 869, 1022, 1207, 1776)	- (446, 628, 846, 1846)
ABA	ABRE	3	3	- (321, 622, 1248)	- (328, 762, 1394)
Auxins	AUX response	1	1	-397	-468
Gibberellins	GARE	1	1	-417	-415
Ethylene	ERE	1	1	-987	-984
Salicylic acid	SA response	1	1	-1313	-1321

*Fc, *Fragaria chiloensis*; *Fv*, Fragaria vesca.*

**FIGURE 3:**
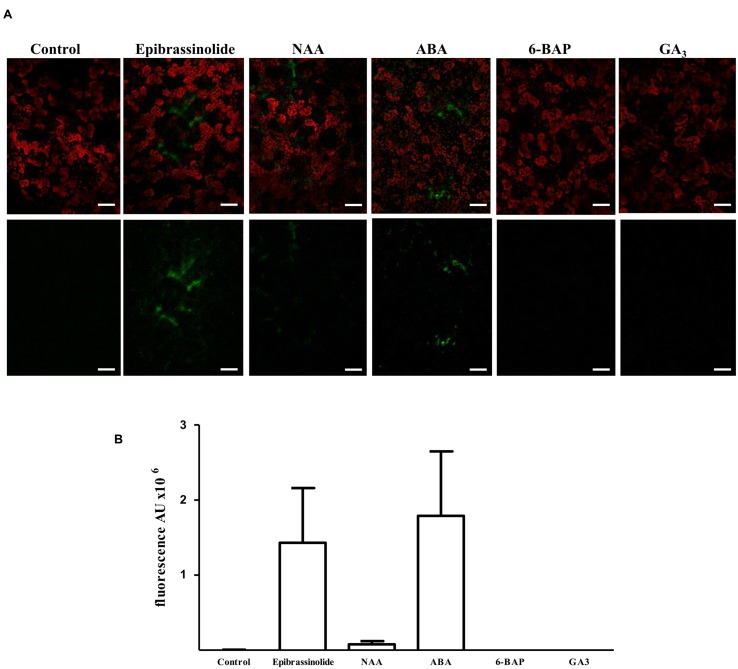
**Response of the promoter of *FcLDP1* to hormonal treatments.** The 1.9 kb promoter of *FcLDP1* was cloned upstream of GFP in pKWSF7.0, and transiently transformed using *Agrobacterium tumefaciens* into tobacco. Three days post-infiltration, hormones were applied for 60 min, and GFP fluorescence monitored by confocal microscopy. **(A)** Brassinosteroid (10 μM epibrassinolide), auxin (10 μM NAA), ABA (100 μM), cytokinin (10 μM 6-BAP), and gibberellins (1 μM GA_3_) were applied. Scale bar: 50 μm. **(B)** The fluorescence intensities of five representative images were quantified using ImageJ software in arbitrary units (AU). Error bars represent standard errors.

In order to assess the role that hormones believed to act during non-climacteric fruit development have on *FcLDP1* expression, two approaches were followed; the effect of exogenous hormone applications on promoter activity in tobacco, and on *FcLDP1* transcript levels in white strawberries. First, to evaluate the regulatory capacity of the promoter, the isolated sequence was fused to the GFP coding sequence and then transiently expressed in tobacco leaves (Yang et al., 2000; Liu et al., 2011). After 3 days, the untreated infiltrated leaves were analyzed by confocal microscopy; no fluorescence was observed, indicating that no basal promoter activity is detectable. Subsequently, infiltrated leaves were sprayed with 24-epibrassinolide and observed over time. This hormone generated a marked increase in fluorescence (**Figures 3A,B**). This result is consistent with the highest levels of *FcLDP1* transcripts in C1 fruits which possess the greatest levels of this hormone (**Figures 1A,C**), and suggests that the REs involved in brassinosteroid responsiveness present in the promoter are indeed functional. Specifically, it is known that BES1 induces transcription of brassinosteroid-responsive genes via E-box motifs (Yin et al., 2005). The presence of E-box motifs within 1800 nucleotides upstream of the start codon in the putative promoter region of *FcLDP1* (**Figure 2**), together with the similar expression profiles of *FcBES1* and *FcLDP1* (**Figures 1A,D**) is consistent with *FcLDP1* being a brassinosteroid responsive gene, possibly mediated by the BES1 transcription factor.

Given that hormones may interact and influence fruit development in different ways (Ozga and Reinecke, 2003), and taking into consideration the presence of other hormonal REs in the *FcLDP1* promoter (**Figure 2**), we applied other hormones to the transiently transformed tobacco leaves and monitored GFP-fluorescence after 1 h. Application of NAA slightly induced transient GFP expression (**Figures 3A,B**). This hormone is essential for the development of *F. × ananassa*, where it is synthesized primarily in the achenes (Aharoni et al., 2002). During the later stages of development of *F. × ananassa*, several authors have reported a sustained increase in the accumulation of ABA (Chai et al., 2011; Jia et al., 2011; Symons et al., 2012). Our results show an induction of promoter activity after application of this hormone, which indicates that at least one of the three ABRE motifs in the promoter could be functional (**Figures 3A,B**). However, not all putative hormone REs were found to be functional. There was no induction of GFP fluorescence after gibberellin (GA_3_) or cytokinin (6-BAP) applications (**Figures 3A,B**). Gibberellins are present in higher concentrations in the early stages of development of *F. × ananassa* fruits, and they are involved in the final size of the fruit because REs to this hormone influence receptacle size (Csukasi et al., 2011). Although the promoter of *FcLDP1* presents one GARE motif, this appears insufficient to trigger a response to this hormone, because a coupled CARE element, essential for gibberellin action, is absent (Sutoh and Yamauchi, 2003). On the other hand, cytokinins are mainly associated with vegetative tissue, and even though they have been proposed to influence non-climacteric sweet grape development (Böttcher et al., 2015), the absence of corresponding REs in the white strawberry promoter under study, may explain the lack of influence of this hormone over promoter activity. In addition, the distance between the REs and the transcription start site also alters the potential for a hormone to control gene expression (Shen and Ho, 1995).

**FIGURE 4:**
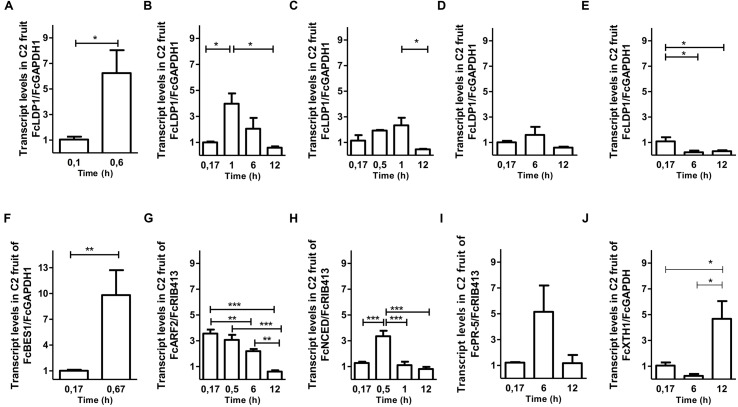
**Effect of hormonal treatments on *FcLDP1* transcript levels.** Effect of 10 μM epibrassinolide **(A)**, 1 mM ABA **(B)**, 1 mM auxin **(C)**, 2 g/L Ethrel **(D)**, and 100 μM methyl jasmonate **(E)** on *F. chiloensis* C2-stage fruits by qRT-PCR. *FcGAPDH* and *FcRIB413* transcript levels were used as a normalizer. *FcBES1* **(F)**, *FcARP2* **(G)**, *FcNCED* **(H)**, *FcXTH* **(I)**, and *FcPR5* **(J)** were used as a positive control in their respective hormonal treatments. The error bars show standard errors and asterisks indicate statistical differences using a Student’s *t*-test (*p < 0.05; **p < 0.01; ***p < 0.001; **A,F**) or one-way ANOVA and Tukey tests (*p < 0.05; **p < 0.01; ***p < 0.001; **B–E**, **G–J**).

Second, to determine whether the responsiveness of the *FcLDP1* promoter to analyzed hormones is also reflected in the strawberry itself, the transcript abundance of *FcLDP1* was quantified directly in stage C2 fruits. The C2 stage was chosen, as *FcLDP1* expression and brassinosteroid levels had yet to peak (**Figures 1A,C**). At this stage, various metabolic changes occur and there is a 72% loss of firmness between stages C2 and C3 (Opazo et al., 2010). As fruit development is a complex process that involves changes in color, texture, flavor, and aroma, non-climacteric fruits such as *F. × ananassa*, present a program of development and maturation guided by different hormones, including auxin, gibberellin, cytokinins, brassinosteroids, ABA, and ethylene (Symons et al., 2012). These hormones were applied to white strawberry fruits as described in Section “Materials and Methods”, and the expression of known response genes was monitored to verify the effectiveness of each treatment (**Figure 4**). Brassinosteroid treatment at stage C2 leads to a significant increase in transcript levels of *FcLDP1* (**Figure 4A**). In non-climacteric fruit, from flowering and fruit set, up to the first stages of fruit development, levels of brassinosteroids are high (Chai et al., 2013). Moreover, exogenous applications of this hormone in grape and *F. × ananassa* promote ripening, whereas treatment with brassinazole, a blocker of brassinosteroid perception, delays this process (Symons et al., 2006; Chai et al., 2013). Similarly, auxins have a higher concentration during the early stages of development favoring fruit growth by cell division and expansion in unripe fruits, yet toward maturation, auxin levels fall in *F. × ananassa* fruits (Aharoni et al., 2002; Fait et al., 2008; Pattison and Catalá, 2012). In this study, the application of NAA, a synthetic auxin leads to an increase in levels of *FcLDP1* transcripts, with a peak at 60 min and decreasing toward 12 h after applying the treatment (**Figure 4B**). On the other hand, application of 1 mM ABA generated a decrease in transcript levels of *FcLDP1* after 1 h post-treatment (**Figure 4C**). Ethylene is a key hormone in climacteric fruit ripening and although in non-climacteric fruit it is present in lower concentrations, *F. × ananassa* mutants in the perception of ethylene show temporal affects in phenylpropanoid metabolism, cell wall degradation, and aroma (Merchante et al., 2013). However, this phytohormone treatment did not produce changes in transcript levels of *FcLDP1* in white strawberries (**Figure 4D**). Finally, methyl jasmonate applications resulted in a decrease in transcript levels after 6 and 12 h (**Figure 4E**). In *F. × ananassa*, it has been observed that applications of this hormone at the green C2 stage induce anthocyanin biosynthesis and degradation of chlorophyll associated with increased accumulation of ethylene (Pérez et al., 1997). In all cases after hormone applications to C2 fruits, the changes in *FcLDP1* transcript levels occur within a short time frame, including a sixfold increase after just 30 min in the case of brassinosteroid treatment. While *FcLDP1* transcript levels increase approximately twofold between stages C2 and C3 (**Figure 1A**), this transition takes approximately 1 week (Salvatierra et al., 2013). Therefore, the most-likely reason for raised *FcLDP1* expression is due to the action of hormone-activated transcription factors on the accessible regions of the *FcLDP1* promoter, and not to the progressive ripening of the fruits during the course of the hormone application experiments.

In *F. × ananassa* fruits, high levels of auxins, brassinosteroids, gibberellins, and methyl jasmonate are found in the initial stages of development (Aharoni et al., 2002). Variations in hormonal contents, particularly in ABA and ethylene, guide the subsequent development of the fruit, leading ultimately to fruits with a greater size, decreased acidity, raised sugar content, loss of chlorophyll, and reduced firmness. In *F. chiloensis*, we analyzed the content of brassinosteroids, which presents two peaks at the C1 and C3 stages. The presence of brassinosteroids induces the expression of a cascade of genes that trigger the development of fruits (Fu et al., 2008). Therefore, considering that *FcLDP1* transcripts accumulate when there are greater brassinosteroid levels, and that it responds transcriptionally to this hormone, it is highly plausible that FcLDP1 plays a role in processes regulated by brassinosteroids during fruit development. For example, SYP2-family syntaxins including AtSYP24 are involved in vasculature development (Shirakawa et al., 2010), and FcLDP1 could play a similar role in the development of the vasculature connections within receptacles, or between achenes and receptacles. Alternatively, as a NDR1/HIN1-like10 protein, it could be involved in pathogen resistance in white strawberry fruits. However, as transcript levels and promoter activity also respond to synthetic auxin, methyl jasmonate and ABA, but not ethylene, it is possible that the function of this gene is also associated with cell division and expansion according to the timing and hormones that induce expression. To test these possibilities, functional characterization of FcLDP1 is required. Nevertheless, the substantial genetic redundancy within the SYP2 family (Shirakawa et al., 2010) and the present lack of characterized insertion lines in *AtSYP24* (At1g32270) and *AtNDR1/HIN1-like10* (At2g35980) pose challenges if the heterologous expression and complementation strategy were to be pursued. Moreover, the generation of *F. chiloensis* mutants, or over-expressing lines for the study of *FcLDP1* (and other genes) is limited due to the current dearth of stable genetic transformation protocols for this specie, compounded by the extended life cycle and octoploidy of the white strawberry. The use of transient transformation, using for example an RNAi-based approach (Salvatierra et al., 2013) could therefore provide a potential alternative means for determining the effects in the shorter term of reducing *FcLDP1* expression.

## Author Contributions

AE designed and performed experiments, and wrote the manuscript. RC performed experiments, GZ performed experiments. RH provided data, MM-L provided data, LN designed experiments and wrote the manuscript. MH designed experiments and wrote the manuscript.

## Conflict of Interest Statement

The authors declare that the research was conducted in the absence of any commercial or financial relationships that could be construed as a potential conflict of interest.

## Footnotes

1 www.rosaceae.org.

2 blast.ncbi.nlm.nih.gov.

3 plantpan2.itps.ncku.edu.tw.

4 http://biotools.umassmed.edu/bioapps/primer3_www.cgi

## Abbreviations

ABA: abscisic acid
FcLDP1: *Fragaria chiloensis* LEA domain protein 1
FvLDP1: *Fragaria vesca* LEA domain protein 1
NAA: 1-naphthaleneacetic acid.
