# Supplementary material for: FcLDP1, a Gene Encoding a Late Embryogenesis Abundant (LEA) Domain Protein, Responds to Brassinosteroids and Abscisic Acid during the Development of Fruits in Fragaria chiloensis
Source: Front Plant Sci. 2016 Jun 14;7:788. doi: 10.3389/fpls.2016.00788 (PMC4905986; doi:10.3389/fpls.2016.00788)
Supplement: Supplementary file 1 [file Table_1.DOCX]

**Supplementary Table S1. Oligonucleotide primers used for PCR and RT-qPCR**

| **Primer** | **Sequence (5'-3')** | **Amplicon (bp)** | **Reference** |
| --- | --- | --- | --- |
| **Fv-LDPf** | CCATGTCGGAGAAACAAGCAGG | 708 | This study |
| **Fv-LDPr** | CATGGATGACCAGCTAGATGGCT |  |  |
| **Fv-pLDPf** | CCTCCAGGCGATATCTTTGA | 1924 | This study |
| **Fv-pLDPr** | CCTGCTTGTTTCTCCGACAT |  |  |
| **Fc-qLDPf** | CTCACCATCCGAAACCCTAA | 102 | This study |
| **Fc-qLDPr** | GGTGAGAGTGATCGTGCTGA |  |  |
| **Fa-qGapdhf** | TCCATCACTGCCACCCAGAAGACTG | 132 | (Pimentel *et al*., 2010) |
| **Fa-qGapdhr** | AGCAGGCAGAACCTTTCCGACAG |  |  |
| **Fa-qRIB413f** | ACCGTTGATTCGCACAATTGGTCATCG | 149 | (Amil-Ruiz *et al.,* 2013) |
| **Fa-qRIB413r** | TACTGCGGGTCGGCAATCGGACG |  |  |
| **Fv-qBes1f** | GGAGGCTCTCGAATGTGGAC | 71 | This study |
| **Fv-qBes1r** | TGATCAGAGCCTGCTGCAAT |  |  |
| **Fa-qNCEDf** | GTTCAAGCTGCAGGAGATGA | 126 | This study |
| **Fa-qNCEDr** | GGCGACTCAACCCAGATTAT |  |  |
| **Fv-qARF2f** | AAGTCTACTGCACGACCAGC | 92 | This study |
| **Fv-qARF2r** | CCCGAGGGCATCCAGTTATC |  |  |
| **Fc-XTH1f** | AATGGCTTCTTCCCAGCAATGTACT | 109 | (Opazo et al., 2010) |
| **Fc-XTH1r** | GTTTCTTCCGAATGGTACAGACACG |  |  |
| **Fc-qPR5f** | CAAGGAGCCAACAAACAGGTCA | 200 | (González et al., 2013) |
| **Fc-qPR5r** | CCACCGTCAGCTACGATGTTG |  |  |
