# Supplementary figures and images for: FcLDP1, a Gene Encoding a Late Embryogenesis Abundant (LEA) Domain Protein, Responds to Brassinosteroids and Abscisic Acid during the Development of Fruits in Fragaria chiloensis
Source: Front Plant Sci. 2016 Jun 14;7:788. doi: 10.3389/fpls.2016.00788 (PMC4905986; doi:10.3389/fpls.2016.00788)

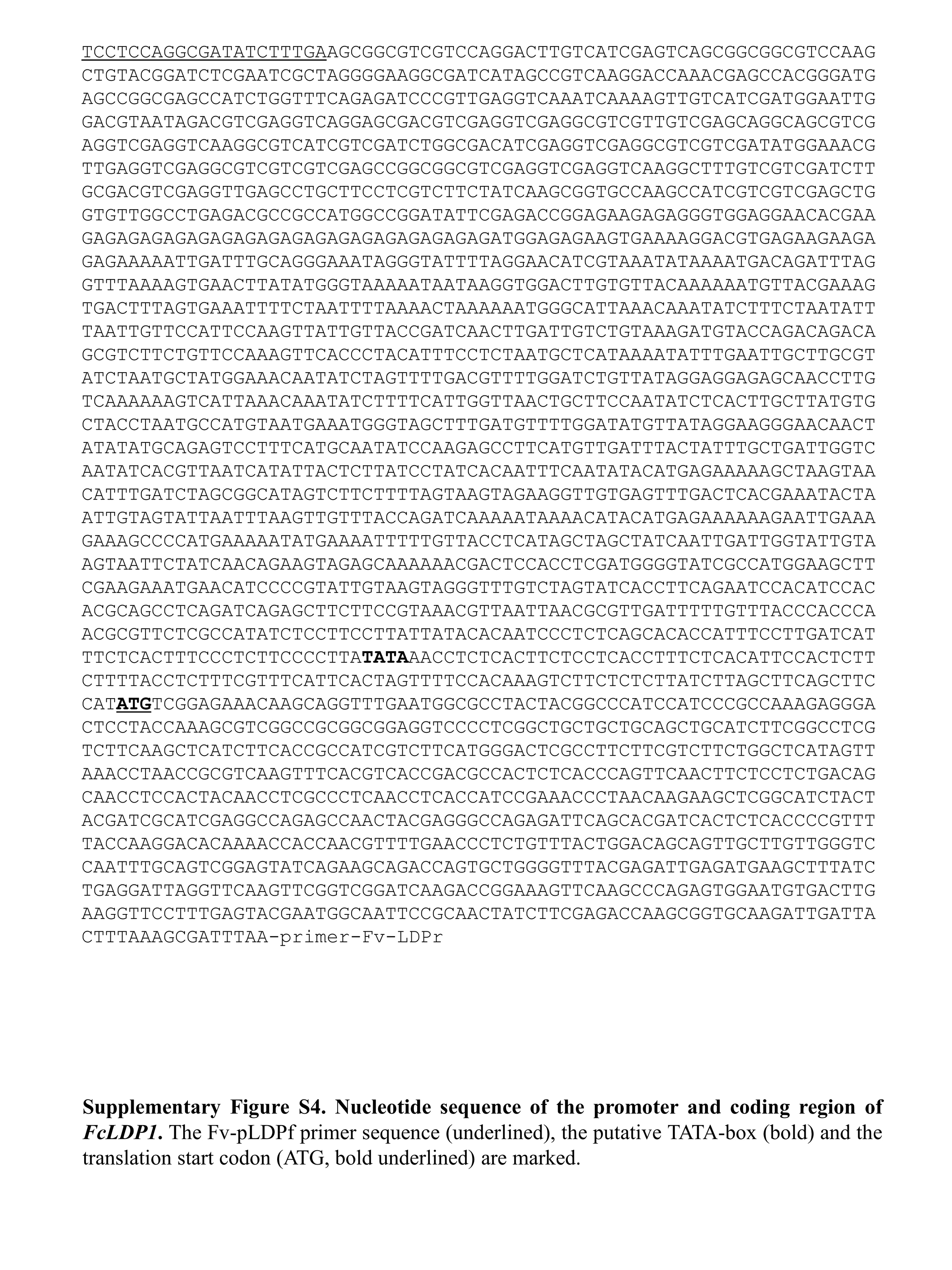

Supplement: Supplementary file 5 [file Image_4.JPEG]
